# Myostatin Levels in SMA Following Disease‐Modifying Treatments: A Multi‐Center Study

**DOI:** 10.1002/acn3.70070

**Published:** 2025-05-14

**Authors:** Fiorella Piemonte, Sara Petrillo, Anna Capasso, Giorgia Coratti, Adele D'Amico, Michela Catteruccia, Maria Carmela Pera, Concetta Palermo, Marika Pane, Emanuela Abiusi, Gianpaolo Cicala, Marianna Villa, Chiara Bravetti, Chiara Arpaia, Agnese Novelli, Salvatore Falqui, Stefania Fiori, Giulia Napoli, Silvia Baroni, Francesco Danilo Tiziano, Enrico Bertini, Giacomo Comi, Stefania Corti, Eugenio Mercuri

**Affiliations:** ^1^ Department of Neurosciences, Unit of Neuromuscular and Neurodegenerative Disorders Bambino Gesù Children's Hospital, IRCCS Rome Italy; ^2^ Pediatric Neurology Unit Catholic University Rome Italy; ^3^ Centro Clinico Nemo, U.O.C. Neuropsichiatria Infantile Fondazione Policlinico Universitario Agostino Gemelli IRCCS Rome Italy; ^4^ Child Neuropsychiatry Unit, Department of Medicine and Surgery University of Parma Parma Italy; ^5^ Section of Genomic Medicine, Department of Life Sciences and Public Health Catholic University of Sacred Heart Rome Italy; ^6^ Department of Basic Biotechnological Sciences, Intensive Care and Perioperative Clinics Catholic University of Sacred Heart Rome Italy; ^7^ Unit of Chemistry, Biochemistry and Molecular Biology “A. Gemelli” Hospital Foundation IRCCS Rome Italy; ^8^ Complex Unit of Medical Genetics Department of Laboratory and Hematologic Sciences, Fondazione Policlinico Universitario A. Gemelli IRCCS Rome Italy; ^9^ SSD Neuromuscular and Rare Diseases Department of Neurosciences and Mental Health, Fondazione IRCCS ca' Granda Ospedale Maggiore Policlinico Milano Italy; ^10^ Dino Ferrari Centre, Department of Pathophysiology and Transplantation (DEPT) Università Degli Studi di Milano Milano Italy

**Keywords:** biomarkers, disease‐modifying therapies, myostatin, neuromuscular disorders, spinal muscular atrophy, treatment response

## Abstract

**Objective:**

This study investigated myostatin levels in SMA patients receiving disease‐modifying therapies (DMTs) to understand their relationship with treatment duration and functional status.

**Methods:**

Our study includes both cross‐sectional and longitudinal analyses of myostatin levels in treated SMA patients. The longitudinal cohort included 46 treatment‐naive patients assessed at baseline and 12 months post‐treatment. Myostatin levels were measured using ELISA. Age‐matched controls (*n* = 89) were included for comparison. The cross‐sectional study included 128 patients with variable durations of treatment (from 0.4 to 7.2 years). In both cohorts, myostatin levels were correlated with SMA type, functional status, and clinical outcomes.

**Results:**

Baseline myostatin levels were significantly lower than controls (*p* < 0.001), except during the neonatal period in presymptomatic patients. After 12 months of treatment, there were no significant changes compared to baseline levels (*p* = 0.1652). The only substantial changes were observed in presymptomatic neonates, who showed a reduction of myostatin despite treatment intervention. There was a significant correlation between myostatin levels, functional status, and SMA type both in the cross‐sectional and longitudinal groups.

**Interpretation:**

This study demonstrates lower myostatin levels in SMA patients compared to controls. The association between myostatin levels, functional status, and SMA type suggests its possible role as a disease severity biomarker. The utility of myostatin as a biomarker for DMT response remains controversial; while we observed no significant increase in myostatin levels following treatment, we also did not observe the progressive reduction previously reported in untreated patients.

## 1 Introduction

Myostatin, a member of the transforming growth factor β (TGF‐β) family, functions as a negative regulator of muscle growth through activin receptor complexes. The observation of increased muscle mass with a supermuscled phenotype in myostatin‐knockout mice and across multiple species, including dogs, cattle, and humans [1, 2, 3, 4, 5], has generated considerable interest in identifying molecules that could inhibit the myostatin signaling pathway. Particular interest has been devoted to possible treatments of muscle atrophy in neuromuscular disorders that can be due to primary muscle involvement or secondary to denervation.

Initial clinical investigations of myostatin inhibitors in muscular dystrophies, particularly Duchenne muscular dystrophy (DMD), yielded disappointing results [6, 7]. A trial using ACE‐031 by Acceleron Pharma (NCT01099761) showed some statistically significant changes from baseline in the lean body mass by DXA and some trends for increased 6‐min walk distance (6MWD) favoring treated versus placebo patients [8]. The results of the trial were limited by safety issues and by the small sample size, but it was felt that other molecules and a different study design may generate better results with fewer adverse events. Subsequent studies in DMD, using domagrozumab (NCT02310763), a recombinant monoclonal immunoglobulin antibody subclass 1 (IgG1) [9] and G6206 (BMS‐986089/Talditercept alpha)/R07239361 (NCT03039686) however, also failed to achieve statistical significance [6]. The recent phase III study using domagrozumab showed promising imaging findings, with increased thigh muscle volume and decreased fat fraction in treated patients, but these changes did not translate into meaningful clinical improvements [9, 10].

The biology, the study designs, and results of the previous trials and provided possible explanations have recently been analyzed [6, 7]. The authors suggested that the total levels of myostatin and downregulation of myostatin expression obtained from the mdx mouse studies may not have been an appropriate model as in humans circulating myostatin is much lower than in mice and there is also a different expression of downregulation of myostatin expression. Another study, exploring myostatin levels in patients with various neuromuscular disorders, also showed very decreased myostatin levels in muscle wasting and atrophying conditions, suggesting that baseline myostatin levels should be carefully evaluated when designing a study [11]. All these findings led to skepticism about the role of myostatin inhibitors in primary muscle disorders.

The advent of disease‐modifying therapies (DMTs) in neuromuscular disorders has revived interest in myostatin inhibitors. This is particularly true for spinal muscular atrophy (SMA), a progressive motoneuron disorder caused by mutations in the SMN gene, where muscle atrophy is secondary to motoneuron loss and SMN protein deficiency [12]. Studies of type I SMA patients, conducted before the advent of DMTs, demonstrated low levels of both myostatin and activin receptor, with a reduced therapeutic potential of myostatin inhibitors [11]. The advent of DMTs (DMTs) that partially restore SMN protein has, however, provided new perspectives on a possible use of myostatin inhibitors in combination with the vailable DMTs [13]. There is recent evidence from both studies on animal model and in humans [14, 15] that the SMN upregulation together with myostatin inhibition enhances therapeutic efficacy, resulting in increased survival, improved function, and enhanced muscle mass with an additional positive influence on sensory neural circuits. These findings provided a compelling rationale for investigating the combination of myostatin inhibitors with SMN‐restoring drugs, a strategy currently being explored in multiple clinical trials. Preliminary results from a recent phase II study using Apitegromab in type II and III SMA patients (NCT) have shown encouraging outcomes, with improvements in motor function at 12 months that were sustained at 36 months [16, 17]. The safety and efficacy of Apitegromab and other molecules targeting the myostatin pathway are currently under investigation in ongoing Phase III studies. Other studies, using other molecules such as GYM329, an anti‐latent myostatin sweeping antibody (Roche) (NCT05115110) [18], and Taldefgrobep alfa, a bivalent, humanized, anti‐myostatin adnectin modified with a human IgG1 Fc tail (Biohaven) (NCT05337553) [19] are currently ongoing.

To better understand how new DMTs affect circulating myostatin levels, we conducted two complementary studies: (a) a longitudinal evaluation of myostatin levels in patients initiating treatment during the study period, measuring levels before and after treatment, and (b) a cross‐sectional analysis examining myostatin levels in a cohort of SMA treated patients (types I, II, and III) to identify correlations with treatment duration. This dual approach allowed us to comprehensively assess both the immediate and long‐term effects of treatment on myostatin expression while analyzing levels in relation to SMA type, functional status, and treatment duration.

## 2 Methods

This prospective, multi‐center study was conducted at three Italian centers: Fondazione Policlinico Gemelli (Roma), Ospedale Pediatrico Bambino Gesù (Roma), and Policlinico di Milano (Milano) between May 2020 and August 2023. The study enrolled both symptomatic patients with a confirmed genetic diagnosis of SMA (types I, II, or III) receiving DMTs and presymptomatic patients identified through neonatal screening, which became available at two centers during the study period.

### 2.1 Study Design

The study design incorporated both cross‐sectional and longitudinal components. Patients who initiated treatment after study initiation were enrolled in the longitudinal cohort, undergoing baseline assessment at treatment initiation and follow‐up evaluation at 12 months post‐treatment, with blood samples collected at both time points. Patients already receiving treatment at study initiation were included in the cross‐sectional cohort, completing a single assessment with documentation of treatment type, duration, and blood sampling.

### 2.2 Clinical Assessments

For all participants, we documented SMA type classification (or presymptomatic status), *SMN2* copy number, and functional status, categorizing patients as non‐sitters, sitters, or walkers. Age‐appropriate functional assessments were conducted using standardized scales. For type I SMA patients, we administered the Children's Hospital of Philadelphia Infant Test of Neuromuscular Disorders (CHOP INTEND) and the Hammersmith Infant Neurological Examination (HINE). Type II and III patients were evaluated using the Hammersmith Functional Motor Scale Expanded (HFMSE) and the Revised Upper Limb Module (RULM).

### 2.3 Laboratory Analysis

Myostatin plasma concentrations were measured on 5 mL peripheral venous blood samples collected into 5% EDTA Vacutainer tubes (Becton Dickinson, Rutherford, NY) using a quantitative sandwich ELISA Kit (GDF‐8/Myostatin Quantikine DGDF80, R&D Systems, Minneapolis, USA), according to the manufacturer's recommendations. Optical density measurements were obtained using an EnSpire Multimode Plate Reader (Perkin Elmer, Waltham, MA, USA), and values were expressed as pg/mL.

### 2.4 Control Group

A control group comprising age‐ and sex‐matched individuals without SMA, who had blood samples collected for unrelated medical reasons, was established to determine the profile of myostatin in the general population and evaluate possible age‐related changes.

### 2.5 Ethics Approval

All study participants or their legal guardians, in the case of minors, provided written informed consent or assent as applicable. The study protocol was approved by the institutional review boards of all participating centers.

### 2.6 Statistical Analysis

Statistical analysis included descriptive statistics for demographic and clinical characteristics. Comparisons between patient and control groups were performed using *t*‐tests, while the Wilcoxon test was employed for paired data analysis in the longitudinal cohort. Additional analyses examined correlations between myostatin levels and clinical parameters, including treatment duration and functional status.

## 3 Results

### 3.1 Longitudinal Study

#### 3.1.1 SMA Longitudinal Study Group—Baseline

A total of forty‐two patients were treatment naive at study entry and could contribute with serum and clinical assessments at both baseline (when the treatment started) and after 12 months. Their age at baseline ranged between 0.03 and 71.3 years (*M* = 14.0, SD = 14.5). The cohort included seven presymptomatic patients identified through newborn screening (16.6%), five type I SMA patients (11.90%), seven type II SMA patients (16.6%), and 23 type III SMA patients (54.76%) (Table 1).

**TABLE 1:** Baseline characteristics of the longitudinal study cohort.

	I (*N* = 5)	II (*N* = 7)	III (*N* = 23)	Presymptomatic (*N* = 7)	Overall (*N* = 42)
Sex assigned at birth					
Female	1 (20.0%)	2 (28.6%)	8 (34.8%)	2 (28.6%)	13 (31.0%)
Male	4 (80.0%)	5 (71.4%)	15 (65.2%)	5 (71.4%)	29 (69.0%)
Age					
Mean (SD)	0.700 (0.8)	19.8 (6.0)	19.2 (15.5)	0.214 (0.4)	14.0 (14.5)
Median [min, max]	0.384 [0.1, 2.1]	18.8 [14.2, 29.9]	16.9 [2.1, 71.3]	0.0466 [0.0, 1.2]	12.6 [0.3, 71.3]
*SMN2* copy number					
2	5 (100%)	1 (14.3%)	5 (21.7%)	3 (42.9%)	14 (33.3%)
3	0 (0%)	5 (71.4%)	11 (47.8%)	2 (28.6%)	18 (42.9%)
4	0 (0%)	1 (14.3%)	7 (30.4%)	2 (28.6%)	10 (23.8%)
CHOP INTEND					
Mean (SD)	27.5 (7.55), *N* = 4	—	—	54.7 (6.65)	44.8 (15.2), *N* = 11
Median [min, max]	27.0 [19.0, 37.0]	—	—	53.0 [46.0, 64.0]	50.0 [19.0, 64.0]
HFMSE					
Mean (SD)	47.0 (NA), *N* = 1	11.1 (19.8)	40.3 (19.2)	—	33.9 (22.5), *N* = 35
Median [min, max]	47.0 [47.0, 47.0]	5.00 [2.00, 56.0]	45.0 [2.00, 63.0]	—	40.0 [2.00, 63.0]
Myostatin level (pg/mL)					
Mean (SD)	994 (762)	659 (697)	1020 (722)	2140 (701)	1140 (837)
Median [min, max]	778 [348, 2290]	365 [260, 2220]	773 [251, 3060]	2320 [846, 2940]	787 [251, 3060]

#### 3.1.2 Myostatin Levels

**FIGURE 1:**
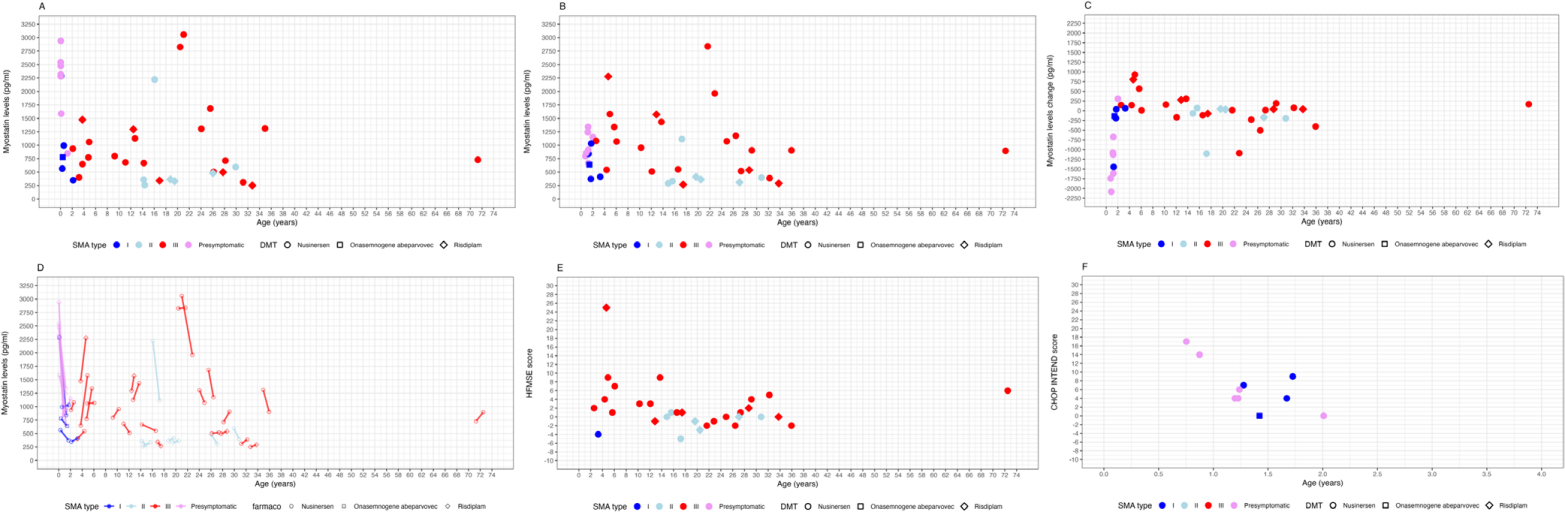
Myostatin levels by SMA type. (Panel A) baseline myostatin levels, (Panel B) 12‐month myostatin levels, (Panel C) changes in myostatin levels at 12 months, (Panel D), individual myostatin trajectories, (Panel E) changes in HFMSE scores, (Panel F) changes in CHOP INTEND scores.

At baseline, myostatin serum concentrations in the study ranged from 251 to 3060 pg/mL (mean: 1140 ± 837 pg/mL) (Figure 1A). Values exceeding 1500 pg/mL were predominantly observed during the neonatal period in presymptomatic patients (6/7 neonatal patients, 85.71%), with only four non‐neonatal patients (9.52%) showing such elevated levels. When stratified by functional status, non‐sitters and sitters showed the lowest mean myostatin levels (663 ± 468 pg/mL, 438 ± 151 pg/mL), followed by ambulant patients (1290 ± 741 pg/mL).

#### 3.1.3 SMA Longitudinal Study Group—12‐Month Changes

Thirty‐one patients completed the HFMSE at baseline and 12 months. The mean change was −1.14 (SD 2.12) for type II (*N* = 7) and 3.26 (SD 5.75) for type III (*N* = 23). Only one patient with type I SMA completed the HFMSE, showing a change of −4.00 points. The overall mean change was 2.03 (SD 5.47) across all patients.

Ten patients completed the CHOP INTEND at baseline and 12 months, with a mean change of 5.00 (SD 3.92) for type I (*N* = 4), 7.50 (SD 6.57) for presymptomatic patients (*N* = 6), and 6.50 (SD 5.54) overall.

#### 3.1.4 Myostatin Levels at Follow‐Up

At follow‐up, serum levels in the study cohort ranged from 268 to 2840 ng/mL (mean = 908 ± 562) (Figure 1B). The changes between baseline and 12‐month follow‐up ranged between −2090 and 930 (mean change: −234 ± 633 ng/mL) (Figure 1C). The largest changes were observed in the children who were presymptomatic (age < 0.5 years) at baseline (mean change: −1390 ± 517 ng/mL).

Excluding the presymptomatic patients in the neonatal period, changes between baseline and 12‐month follow‐up ranged between −1450 and 930 (mean change: −51.1 ± 461 ng/mL).

The Wilcoxon test for paired data comparing the baseline and follow up serum myostatin levels showed no significant difference before and after treatment (*V* = 563, *p* = 0.1652) across the entire cohort and excluding pre‐symptomatic patients (*p* = 0.9022).

A linear mixed‐effects model was fit to assess the relationship between various predictors and myostatin levels over 12 months in patients with an age at baseline greater than 0.5 years (Table 2, Model A). The analysis included time, age at baseline, sex (coded as male/female), functional status (“non sitter,” “sitter,” and “walker”), and SMN2 copy number as fixed effects. Random intercepts were modeled for the grouping factor (subject ID).

**TABLE 2:** Results of the linear mixed model.

Fixed effect	Model A estimate	Model A, *p*	Model B estimate	Model B, *p*
(Intercept)	6.59	0.0000	5.91	< 0.001
Time	0.02	0.6655	−0.04	0.4631
Age at baseline	0.002	0.6713	0.00	0.5089
Sex assigned at birth (male vs. female)	−0.04	0.8386	0.03	0.8630
Functional status at baseline (sitters vs. non sitters)	−0.35	0.4145	0.07	0.8913
Functional status at baseline (walkers vs. non sitters)	0.86	0.0608	1.25	0.0275*
SMA II vs. SMA I	−0.13	0.7644	−0.25	0.3165
SMA III vs. SMA I	−0.10	0.8224	−0.40	0.1712
Presymptomatic vs. SMA I	−0.14	0.8240	—	—
3 *SMN2* copies vs. 2 *SMN2* copies	−0.31	0.1996	0.14	0.7917
4 *SMN2* copies vs. 2 *SMN2* copies	−0.38	0.1920	0.07	0.8927
12‐month HFMSE change	—	—	0.03	0.0029*

* *p*‐value < 0.05.

A second linear mixed‐effects model was fit to assess the relationship between various predictors and myostatin levels over 12 months in patients with an age at baseline greater than 0.5 years and with the HFMSE performed at both timepoints (Table 2, Model B). The analysis included time, age at baseline, sex (coded as male/female), functional status (“non sitter,” “sitter,” and “walker”), HFMSE changes, and *SMN2* copy number as fixed effects. Random intercepts were modeled for the grouping factor (subject ID). For both models, myostatin levels were transformed using a log transformation to address the positive skewness observed in the data.

#### 3.1.5 Control Group Versus SMA Study Group

The control group included 89 individuals matched for age and sex with the SMA longitudinal study group (Table 3).

**TABLE 3:** Comparison between control and SMA study groups.

	Control group (*N* = 89)	SMA group (*N* = 178)	*p*
Age			
Mean (SD)	20.9 (13.6)	18.8 (18.1)	0.296
Median [min, max]	20.9 [0.417, 55.1]	14.2 [0.0301, 72.5]	
Sex assigned at birth			
Female	48 (53.9%)	82 (46.1%)	0.279
Male	41 (46.1%)	96 (53.9%)	

Myostatin serum levels in the control group ranged between 224 and 7000 ng/mL (mean: 2330 ± 1420). A Pearson's product–moment correlation was conducted to examine the relationship between age and myostatin. The results showed a weak, positive correlation that was not statistically significant, *r* (87) = 0.17, *p* = 0.11. The 95% confidence interval for the correlation ranged from −0.04 to 0.37. Values above 2000 ng/mL were found in more than 50% of the controls, irrespective of age (Figure S1).

A Welch's two‐sample *t*‐test was conducted to compare the means of the SMA study group (*M* = 1522.73) and the Control group (*M* = 2311.98). The results indicated a statistically significant difference between the groups, *t* (216.72) = −4.13, *p* < 0.001, with a 95% confidence interval for the difference in means ranging from −1166.03 to −412.47.

Subgroup analysis of the neonatal period showed comparable levels between SMA individuals (*M* = 2143.30) and controls (*M* = 2993.33) *t* (2.74) = −1.10, *p* = 0.358, with a 95% confidence interval for the difference in means ranging from −3443.62 to 1743.55.

#### 3.1.6 Cross‐Sectional Cohort

The cross‐sectional component included 128 patients with varying disease severity and treatment exposure. Of these 128, 27 were type I (1 walker, 13 sitters, 13 non sitters), 35 type II (27 sitters and 8 non sitters) and 60 type III (3 non sitters, 24 sitters and 33 walkers). Six patients were asymptomatic (1 non‐sitter, 2 sitters, 3 walker). Their age ranged between 0.75 and 72.5 years (mean = 19.5, SD = 18.1). Figure 1 shows myostatin levels in relation to SMA type and age.

The myostatin levels (pg/mL) ranged between 200 and 10,100 (mean = 1610, SD = 1880).

**FIGURE 2:**
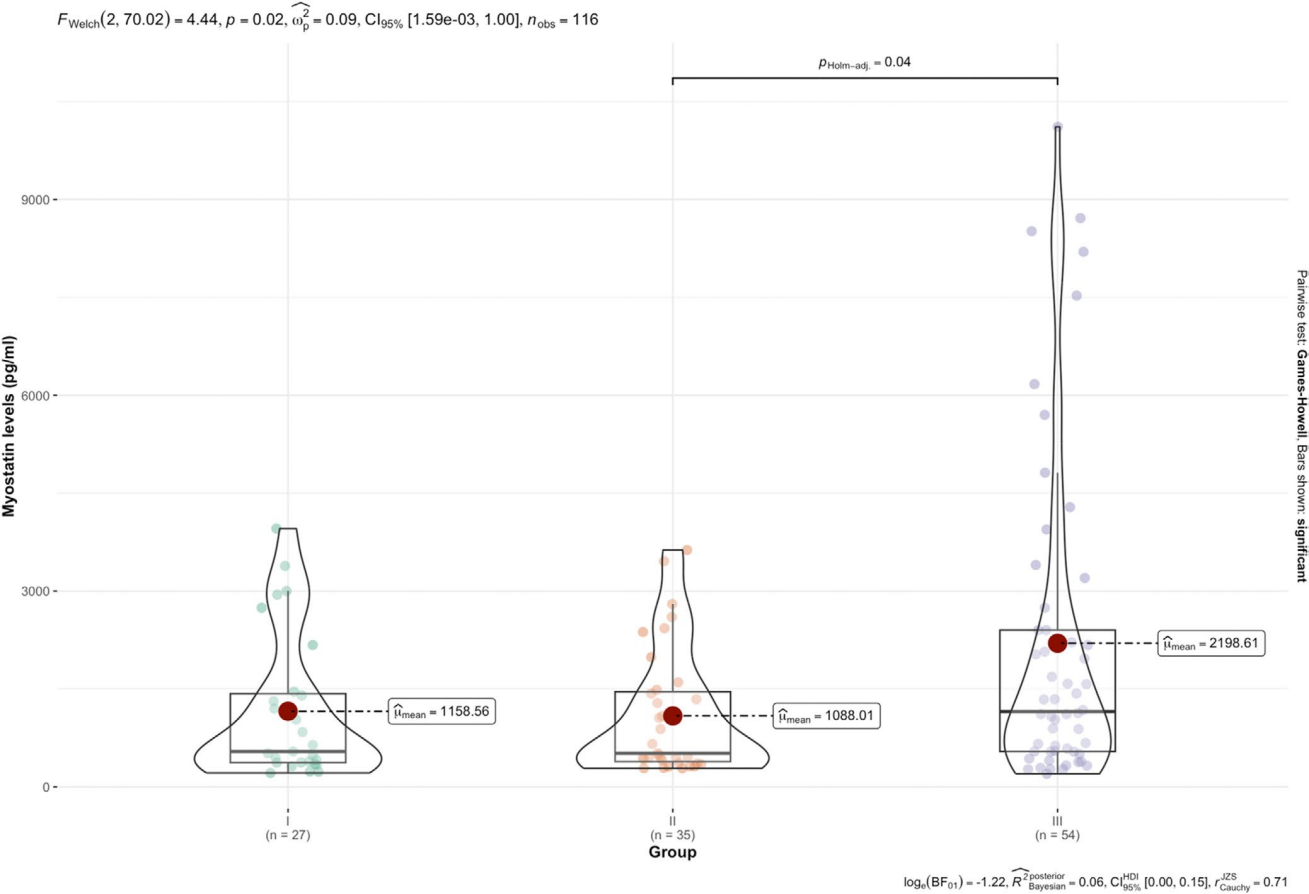
Myostatin levels in relation to functional status.

An analysis was conducted to evaluate the relationship between SMA type and myostatin levels. The results of the Welch's *F*‐test indicated a significant difference between the groups [*F* (2, 70.02) = 4.44, *p* = 0.02] (Figure 2).

**FIGURE 3:**
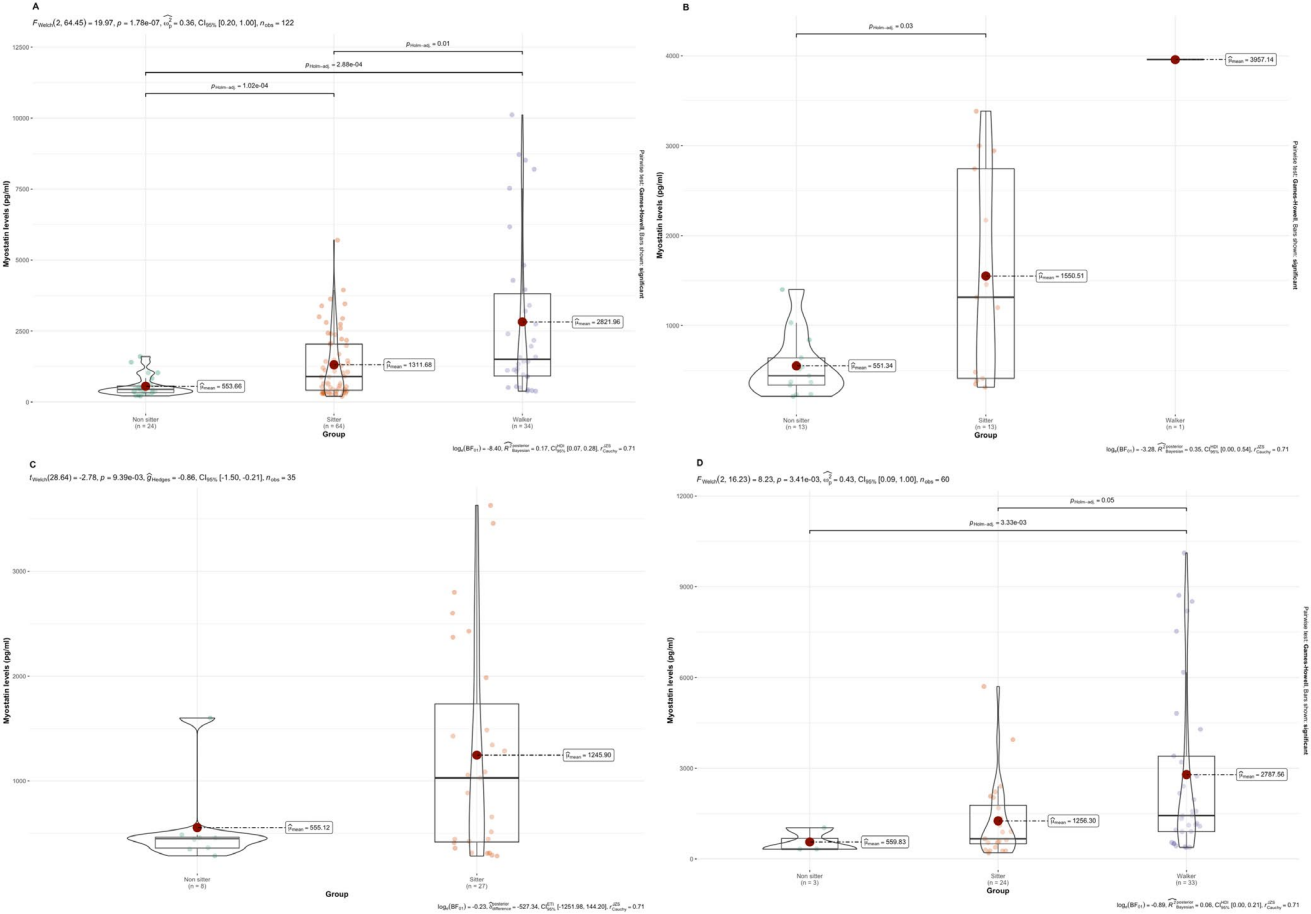
Myostatin levels in relation to SMA type and functional status. (Panel A) overall, (Panel B) SMA I, (Panel C) SMA II, (Panel D) SMA III.

An analysis was conducted to evaluate the relationship between functional status (non‐sitter, sitter, and walker) and myostatin levels. The results of the Welch's *F*‐test revealed a significant difference in myostatin levels across functional status [*F* (2, 58.06) = 20.47, *p* = 1.87 × 10^−7^] (Figure 3A).

In type I SMA, post hoc comparisons using the Holm correction showed a significant difference in myostatin levels between non‐sitters and sitters (*p* = 0.03, Figure 3B).

For type II SMA, the Welch's *t*‐test indicated a significant difference in myostatin levels across the functional status [*t* (28.64) = −2.78, *p* = 9.39 × 10^−3^] (Figure 3C).

Finally, in type III SMA, a significant difference in myostatin levels was observed [*F* (2, 17.5) = 8.80, *p* = 2.26 × 10^−3^] (Figure 3D).

A Pearson's product–moment correlation was conducted to assess the relationship between treatment duration and myostatin levels. The analysis revealed a very weak, almost negligible correlation [*r* = 0.02, *t* (126) = 0.27, *p* = 0.785], indicating no significant relationship between these two variables. The 95% confidence interval for the correlation coefficient ranged from −0.15 to 0.20, further supporting the lack of a meaningful association.

**FIGURE 4:**
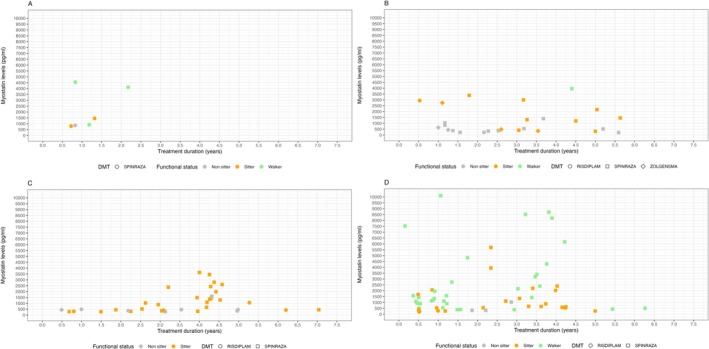
Myostatin levels in relation to SMA type, functional status and duration of treatment. (Panel A) presymptomatic patients, (Panel B) SMA I individuals, (Panel C) SMA II individuals, (Panel D) SMA III individuals.

Figure 4 shows myostatin levels in relation to SMA type, functional status, and duration of treatment.

An analysis was conducted to evaluate the relationship between *SMN2* copy number and myostatin levels. The results of the Welch's *F*‐test revealed a significant difference in myostatin levels across SMN2 copy number[*F* (2, 43.41) = 8.81, *p* = < 0.001], with a significant difference between 2 *SMN2* copies and 4 *SMN2* copies, and between 3 *SMN2* copies and 4 *SMN2* copies (both *p* < 0.001).

A Pearson's product–moment correlation was conducted to assess the relationship between HFMSE and myostatin levels. There was a moderate, positive correlation between the two variables [*r* = 0.51, *t* (106) = 6.06, *p* < 0.001]. The 95% confidence interval for the correlation coefficient ranged from 0.35 to 0.64, indicating a statistically significant association.

A Pearson's product–moment correlation was performed to examine the relationship between CHOP INTEND and myostatin levels. Results revealed a moderate, positive correlation [*r* = 0.57, *t* (31) = 3.83, *p* = 0.0006]. The 95% confidence interval for the correlation coefficient ranged from 0.28 to 0.76, indicating a statistically significant association.

One hundred individuals were treated with nusinersen, 23 with risdiplam, and 5 with onasemnogene aberparvovec. Of these, 3 switched from nusinersen to onasemnogene aberparvovec, one from nusinersen to risdiplam, and one from risdiplam to onasemnogene aberparvovec.

Figure S2 displays myostatin levels in the risdiplam and nusinersen groups, excluding presymptomatic and type I patients to ensure population balance as they were only treated with nusinersen. After balancing, the two treatment groups were comparable for age and SMA type (*p* > 0.05) but a significant difference was observed in functional status as measured by the HFMSE, with the risdiplam group showing lower scores (median: 4.50, range: 0–62) compared to the nusinersen group (median: 29, range: 0–66; *p* = 0.008, Mann–Whitney *U* test *p* = 0.008).

A Mann–Whitney *U* test revealed a significant difference in myostatin levels between the groups (*W* = 394.50, *p* < 0.001), with patients treated with nusinersen showing higher myostatin levels compared to those treated with risdiplam (median: 1176 vs. 449.61, respectively).

## 4 Discussion

Over the last few years, there has been increasing interest in the role of myostatin as a possible biomarker of disease severity in SMA [11, 20, 21]. Our study provides novel insights into the dynamics of myostatin levels in SMA patients receiving DMTs.

At baseline, before treatment started, SMA patients had significantly lower baseline myostatin levels compared to age‐matched controls, in line with previous reports of decreased myostatin expression in SMA patients [11, 20, 21].

There was a significant difference in myostatin levels according to functional status in the whole cohort and also across all SMA types. The hierarchical pattern of myostatin levels observed across functional categories—lowest in non‐sitters and highest in ambulant patients—likely reflects the relationship between muscle function, muscle structure, and myostatin expression. There was no significant difference according to age, with a weak, positive correlation that was not statistically significant [*r* (87) = 0.17, *p* = 0.11]. These findings are in agreement with other studies also showing that age did not correlate to myostatin levels in the whole SMA cohort [20, 21] with only a negative correlation in type 1 SMA [20]. When analyzing age subgroups, we found that in the neonatal period, the overall myostatin levels appeared to be higher and more similar to the age‐matched controls. Our results, however, were driven by the presence of six presymptomatic newborns identified through neonatal screening who showed higher myostatin levels compared to the newborns who already had clinical signs. The strong correlation between functional status and myostatin levels confirms that interventions with myostatin inhibitors need to be tailored according to patients' functional capabilities.

Our study also provided additional evidence of possible myostatin level changes following treatment with available DMTs. A recent paper reported relatively small changes in myostatin levels before and after treatment with nusinersen in patients with type I, II, and III SMA [20]. Our findings confirm and expand the previous results in a larger cohort, including patients treated with two other DMTs, risdiplam and onasemnogene abeparvovec. The longitudinal analysis exploring the possible changes in myostatin levels after 12 months of treatment confirmed previous findings, with small changes in the whole cohort [20]. The only relevant changes in myostatin levels were observed in presymptomatic patients starting treatment during the neonatal period, in whom myostatin levels at baseline were similar to the controls but showed a consistent reduction of myostatin levels during the first year.

The lack of increase in myostatin levels following treatment was further confirmed by the cross‐sectional analysis, which allowed assessment of myostatin levels in relation to different exposure to treatments in a larger cohort. Although patients with longer exposure in each SMA type showed a trend toward higher values, the correlation with treatment duration was not significant. Because of the cross‐sectional nature of this observation, we cannot exclude that these patients may already have had high values at baseline, as observed in some patients with longitudinal data.

The lack of increase after treatment appears to suggest that available DMTs may have no effect on myostatin levels, and that, at least with a limited follow‐up of 12 months, myostatin regulation operates independently of the clinical changes observed in the same time frame. These findings, however, should be interpreted with caution for several reasons. First, it is not fully understood how serum levels of myostatin relate to muscle levels in neuromuscular diseases such as SMA, and longitudinal data in untreated patients that could be used as comparators are scanty. A recent longitudinal study measuring myostatin levels in untreated patients reported a significant reduction in myostatin levels after a 12 month follow‐up (*p* = 0.021) [21]. One could therefore postulate that the relative stability of myostatin levels in our treated cohort may therefore follow a similar pattern to that observed in clinical studies. Older type I and, even more, types II and III patients treated after age 5 years often show stable scores on functional scales that can be interpreted as a response to treatment only when compared to reduced scores in matched untreated patients [22, 23, 24, 25]. The hypothesis that myostatin level stability may be considered a possible response to treatment is also supported by the association between myostatin and HFMSE changes.

In conclusion, our study demonstrates complex dynamics of myostatin regulation in SMA patients receiving DMTs. One limitation of our study is that although we included individuals treated with all three available DMTs, the information provided on individual drugs is limited. As this was an observational study, collecting real world data based on families' choices of treatment, we were unable to prospectively stratify patients according to treatment. Given that nusinersen was the first approved treatment, patients receiving this therapy represent the majority of those with the longest treatment durations. Conversely, access to risdiplam was initially provided through compassionate use programs, primarily for individuals ineligible for nusinersen [26]. As a result, the risdiplam cohort had significantly lower baseline motor function, as evidenced by markedly reduced HFMSE scores (mean 4.50 vs. 29; *p* = 0.008). Moreover, given the demonstrated strong correlation between HFMSE scores and serum myostatin levels, it is not possible to determine whether the observed statistically significant difference in myostatin levels between treatment groups reflects a true pharmacological effect or is confounded by baseline functional disparities.

Our findings also contribute to a better understanding of myostatin's possible role as a biomarker. While the correlation between myostatin levels and functional status, SMA type, and *SMN2* copies suggests that myostatin expression might serve as a biomarker of disease severity, the longitudinal studies available in patients treated with current DMTs, based on relatively small cohorts, show a relative stability that cannot be easily interpreted without comparing these findings to similar follow up in matched untreated patients.

Finally, our findings also have implications for therapeutic strategies in SMA, especially in presymptomatic patients. The preserved myostatin levels in presymptomatic neonates and their fall after the neonatal period suggest a possible developmental window during which myostatin regulation may still be preserved in presymptomatic patients, with the possibility of enhanced responses from combining SMN‐restoring and myostatin‐targeting therapies. Further studies, including assessments of lean body mass through DXA scans, may help to better understand the relationship between muscle mass and myostatin levels and response to DMTs.

## Author Contributions

Fiorella Piemonte, Sara Petrillo, Anna Capasso, Stefania Corti, Giacomo Comi, Enrico Bertini and Eugenio Mercuri were involved in the conception and design of the study, analysis of data, and drafting a significant portion of the manuscript or figures. Adele D’Amico, Michela Catteruccia, Maria Carmela Pera, Concetta Palermo, Marika Pane, Emanuela Abiusi, Francesco Danilo Tiziano, Enrico Bertini, Giacomo Comi, Stefania Corti and Eugenio Mercuri contributed to the acquisition and analysis of data and provided critical input during manuscript preparation. Gianpaolo Cicala, Marianna Villa, Chiara Bravetti, Chiara Arpaia, Agnese Novelli, Salvatore Falqui, Stefania Fiori, Giulia Napoli and Silvia Baroni contributed to the acquisition and analysis of data. Stefania Corti and Eugenio Mercuri are responsible for the overall content as guarantor. All authors reviewed and approved the final manuscript.

## Conflicts of Interest

Giorgia Coratti reports are part of advisory boards from Biogen S.R.L., Roche, and Novartis outside the submitted work. Maria Carmela Pera is part of advisory boards for Roche outside the submitted work. Marika Pane is part of advisory boards for Biogen S.R.L. outside the submitted work. Enrico Bertini is part of advisory boards for Biogen S.R.L., Roche, PTC, UCB, and Pfizer outside the submitted work. Giacomo Comi is part of advisory boards from Biogen S.R.L., Roche, Scholar Rock, Sarepta, and Novartis outside the submitted work. Stefania Corti is part of advisory boards from Biogen S.R.L., Roche, Scholar Rock, and Novartis outside the submitted work. Eugenio Mercuri is part of advisory boards for Biogen S.R.L., Roche, and Novartis, Scholar Rock, Epirium, and Cytokinetics outside the submitted work. The other authors declare no conflicts of interest.

## Supporting information


**Figure S1.** Correlation between age and myostatin levels. (Panel A) controls; (Panel B) SMA individuals.
**Figure S2.** Myostatin levels between risdiplam and nusinersen individuals.

## Data Availability

The data that support the findings of this study are available from the corresponding author upon reasonable request.
