# Supplementary figures and images for: Myostatin Levels in SMA Following Disease‐Modifying Treatments: A Multi‐Center Study
Source: Ann Clin Transl Neurol. 2025 May 14;12(7):1368–77. doi: 10.1002/acn3.70070 (PMC12257122; doi:10.1002/acn3.70070)

**Supplementary figures**

**Supplementary figure 1**


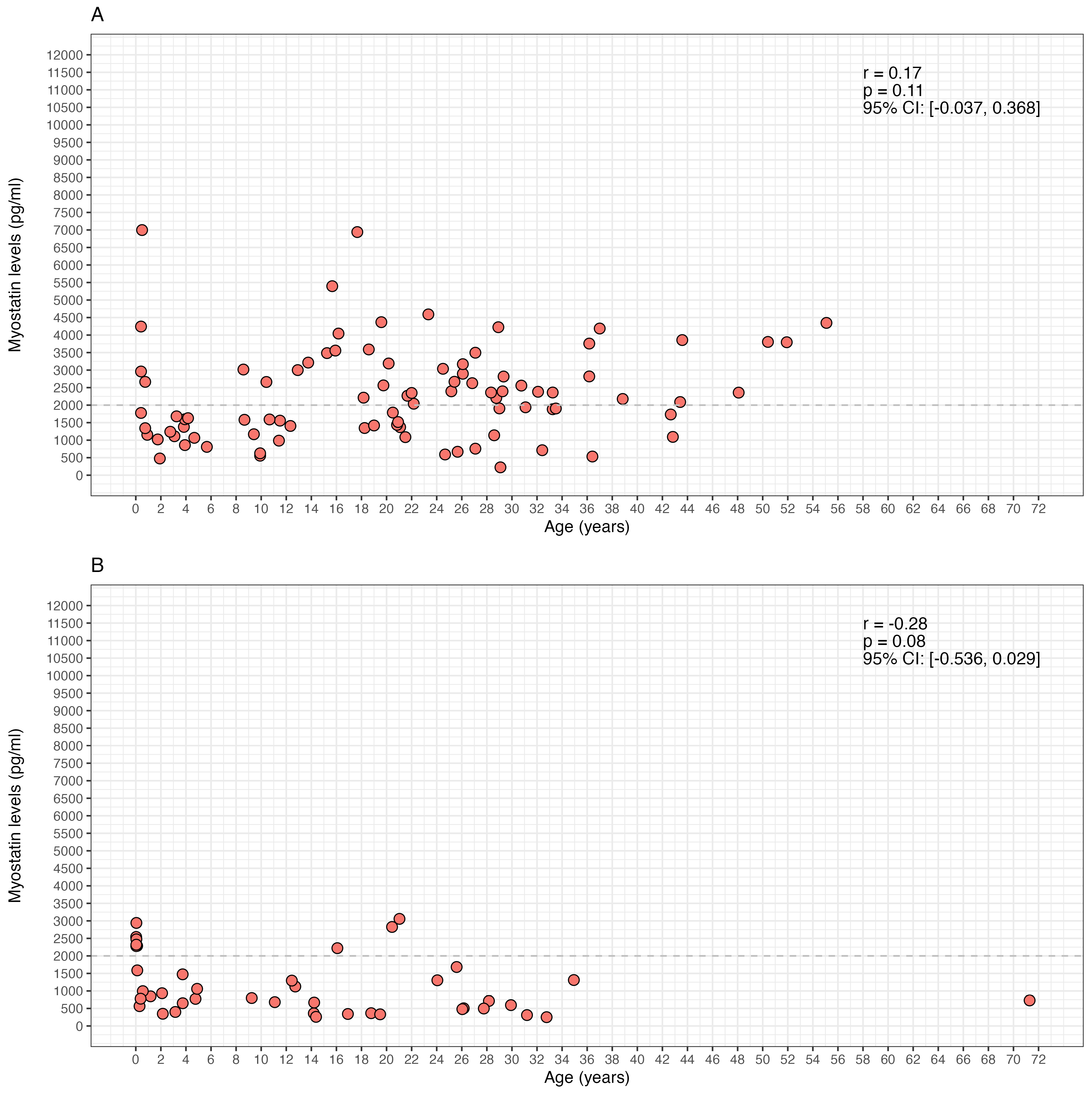


**Supplementary figure 2**

**
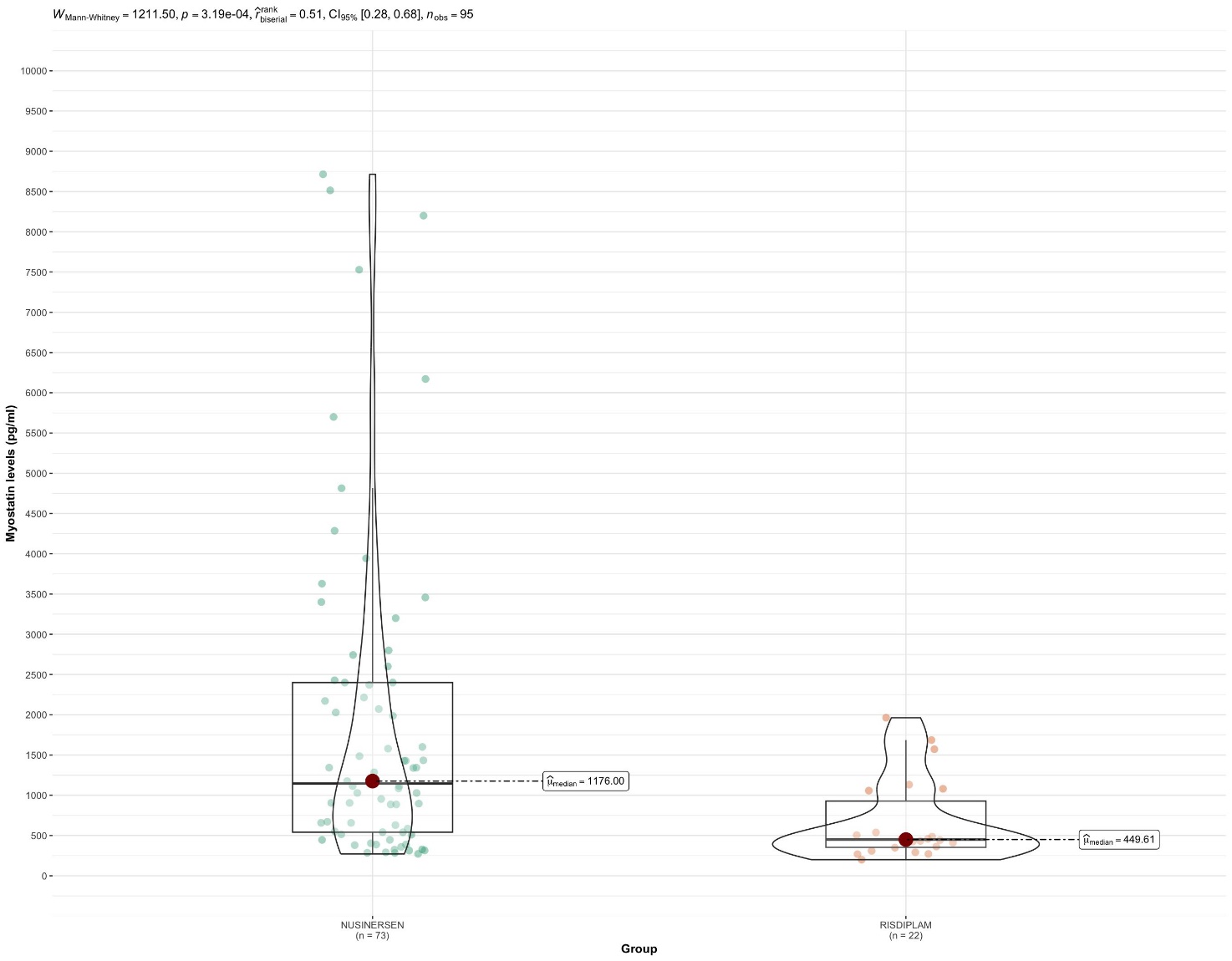
**

Supplement: Supplementary file 1 — Figure S1. Correlation between age and myostatin levels. (Panel A) controls; (Panel B) SMA individuals. Figure S2. Myostatin levels between risdiplam and nusinersen individuals. [file ACN3-12-1368-s001.docx]
